# Temporal Trends and Center Variation in Early Antibiotic Use Among Premature Infants

**DOI:** 10.1001/jamanetworkopen.2018.0164

**Published:** 2018-05-25

**Authors:** Dustin D. Flannery, Rachael K. Ross, Sagori Mukhopadhyay, Alison C. Tribble, Karen M. Puopolo, Jeffrey S. Gerber

**Affiliations:** 1 Center for Pediatric Clinical Effectiveness, Children’s Hospital of Philadelphia, Philadelphia, Pennsylvania; 2 Division of Neonatology, Children’s Hospital of Philadelphia, University of Pennsylvania, Philadelphia; 3 Division of Pediatric Infectious Diseases, Children’s Hospital of Philadelphia, University of Pennsylvania, Philadelphia; 4 Division of Pediatric Infectious Diseases, C. S. Mott Children’s Hospital, University of Michigan, Ann Arbor

## Abstract

**Importance:**

Premature infants are frequently administered empirical antibiotic therapy at birth. Early and prolonged antibiotic exposures among infants without culture-confirmed infection have been associated with increased risk of adverse outcomes.

**Objective:**

To examine early antibiotic use among premature infants over time and across hospitals in the United States.

**Design, Setting, and Participants:**

This retrospective cohort study used a comprehensive administrative database of inpatient encounters from 297 academic and community hospitals across the United States to examine data concerning very low-birth-weight (VLBW) infants (<1500 g), including extremely low-birth-weight (ELBW) infants (<1000 g), who were admitted to the neonatal intensive care unit and survived for at least 1 day. Data collection took place in November 2015 and analysis took place from February 2016 to November 2016.

**Exposures:**

Antibiotic initiation within the first 3 days of age and subsequent antibiotic administration for more than 5 days.

**Main Outcomes and Measures:**

Temporal trends in early antibiotic initiation and duration from 2009 to 2015, and center variation in early antibiotic use from 2014 to 2015.

**Results:**

We identified 40 364 VLBW infants (20 447 female [50.7%]) who survived for at least 1 day, including 12 947 ELBW infants, from 297 centers. The majority of premature infants had early antibiotic initiation (31 715 VLBW infants [78.6%] and 11 264 ELBW infants [87.0%]), and no differences were observed over time in temporal trend analyses (*P* = .12 for VLBW and *P* = .52 for ELBW). The annual risk difference in the proportion of VLBW infants administered early antibiotic therapy ranged from −0.75% (95% CI, −1.61% to 0.11%) to −0.87% (95% CI, −2.04% to 0.30%); in ELBW infants the annual risk difference ranged from −0.34% (95% CI, −1.28% to 0.61%) to −0.38% (95% CI, −1.61% to 0.85%). There was a small but significant decrease over time in the rate of prolonged antibiotic duration for VLBW infants (*P* = .02), but not for ELBW infants (*P* = .22). The annual risk difference in the proportion of VLBW infants with prolonged antibiotic duration ranged from −0.94% (95% CI, −1.65% to −0.23%) to −1.08% (95% CI, −2.00% to −0.16%); in ELBW infants the annual risk difference ranged from −0.72% (95% CI, −1.83% to 0.39%) to −0.75% (95% CI, −1.96% to 0.46%). We also observed variation in early antibiotic exposures across centers. Sixty-nine of 113 centers (61.1%) started antibiotic therapy for more than 75% of VLBW infants, and 56 of 66 centers (84.8%) started antibiotic therapy for more than 75% of ELBW infants. The proportion of VLBW and ELBW infants administered prolonged antibiotics ranged from 0% to 80.4% and 0% to 92.0% across centers, respectively.

**Conclusions and Relevance:**

Most premature infants in this study received empirical early antibiotic therapy with little change over a recent 7-year period. The variability in exposure rates across centers, however, suggests that neonatal antimicrobial stewardship efforts are warranted to optimize antibiotic use for VLBW and ELBW infants.

## Key Points

**Question:**

Have empirical early antibiotic prescribing patterns for premature infants changed over time?

**Findings:**

This multicenter cohort study of more than 40 000 premature infants found that most premature infants received early antibiotic administration and that rates of initiation of empirical early antibiotic therapy did not change from 2009 to 2015. Rates of prolonged antibiotic administration among very low-birth-weight infants decreased slightly, but did not change among extremely low-birth-weight infants.

**Meaning:**

Despite concerns regarding the negative impact of early and prolonged empirical antibiotic use among premature newborns, neonatal clinicians across the United States continue to prescribe empirical antibiotics to the majority of very low-birth-weight infants in the first 3 days of age.

## Teaser

This pharmacoepidemiology study assesses rates and duration of antibiotic use begun within the first 3 days of life in very low-birth-weight and extremely low-birth-weight infants using administrative database data of academic and community hospitals from 2009 to 2015.

## Introduction

Antibiotics are the most common class of medications used in the neonatal intensive care unit (NICU).^1,2^ Very low-birth-weight (VLBW) (birth weight <1500 g) infants are frequently administered empirical antibiotic therapy at birth because they are at risk for early-onset sepsis (EOS). Infection is the cause of approximately one-third of cases of preterm labor and/or premature rupture of membranes,^3,4^ and approximately 1 in 90 VLBW infants is born with culture-confirmed early-onset bacterial infection.^5^ Infection-attributable mortality is 35% among VLBW infants with EOS and as high as 50% among the most premature infants, those born at 22 to 24 weeks’ gestation.^6^ The probability of EOS combined with the clinical instability characteristic of premature newborns leads to high rates of empirical antibiotic treatment: previous studies demonstrate that neonatal clinicians administer antibiotics from birth in 90% to 98% of VLBW and extremely low-birth-weight (ELBW) (birth weight <1000 g) infants.^7,8^

Although antibiotics are administered to protect premature newborns, multiple recent studies suggest that antibiotics have potential risks as well as potential benefits for this population. Early and prolonged antibiotic exposure in premature infants without culture-confirmed infection has been associated with increased mortality as well as increased subsequent risks of necrotizing enterocolitis, invasive fungal infections, antibiotic-resistant infections, bronchopulmonary dysplasia, periventricular leukomalacia, and retinopathy of prematurity.^8,9,10,11,12,13,14,15,16,17,18^ Recent studies have identified declines in antibiotic use rates during hospitalization over time among premature infants in Canadian centers^12^ and variation in antibiotic use rates among all newborns in neonatal centers in California independent of infection burden.^19^ Such variation suggests that some proportion of antibiotic use may be unnecessary. Antimicrobial stewardship efforts have begun to focus on neonatal settings, and national quality improvement initiatives have targeted nonindicated early antibiotic use in VLBW infants.^20,21,22,23^ The impact of such efforts remains unclear. A detailed understanding of how antibiotics are used among specific categories of newborns may help optimize such efforts. The objective of this study was to specifically examine early antibiotic use among VLBW infants in a contemporary and nationally representative database to describe trends over time and across hospitals in the United States.

## Methods

### Data Source and Study Population

We conducted a retrospective cohort study using the Premier Perspective Database (Premier Inc). Premier is a large, comprehensive administrative database of deidentified inpatient encounters from participating hospitals across the United States and has previously been used for research studies in both adult and pediatric populations.^24,25,26,27,28^ The database includes both academic medical centers and community-based hospitals; contains diagnosis and procedure codes, demographic information, and billed services, including pharmacy charges; and is subject to routine validation and quality audits. Deidentified hospital-level information, including teaching status, hospital size, and geographic region, are available. Patient-level daily drug charge data, including drug name, class, dosage, and route, are also available. This study was not considered human subjects research given the fully deidentified nature of the data and was therefore exempted from institutional review board review and requirement for patient consent according to the policy of the Children’s Hospital of Philadelphia institutional review board. The Strengthening the Reporting of Observational Studies in Epidemiology (STROBE) reporting guideline was used in the reporting of this study.

Data collection took place in November 2015 and analysis took place from February 2016 to November 2016. We identified patients in the Premier database between January 2009 and September 2015 with (1) a neonatal All Patient Refined Diagnosis Related Group (APR-DRG) code, (2) patient origin of “born inside this hospital,” (3) admission listed as “inpatient,” (4) NICU room and board charge on the first or second day of admission (to only include infants admitted to the NICU within 24 hours of birth), and (5) birth at a center that cared for more than 20 newborn patients per year (to eliminate infants born at centers that were unlikely to be functional neonatal units). We excluded patients who survived for less than 1 day or had missing birth weight data. Using the APR-DRG codes, we identified VLBW infants and the subset of ELBW infants.

### Study Definitions

We defined the day of birth as the day of hospital admission, given that we only included inborn infants and that VLBW infants are universally admitted to the NICU at birth in the United States. Early antibiotic initiation was defined as a billing charge for at least 1 parenteral antibiotic in the first 3 days of age. Prolonged antibiotic duration was defined as administration of parenteral antibiotics for more than 5 consecutive days after early initiation. This was chosen to align with the definition of prolonged antibiotic duration previously used by a large multicenter neonatal-specific database.^8^ For the analysis of prolonged antibiotic administration, we excluded infants admitted to the NICU for 5 or fewer days (ie, discharged or died ≤5 days of age).

### Statistical Analysis

We calculated the total duration (days) of early antibiotic therapy among all infants in the study sample, the proportions of VLBW and ELBW infants with early antibiotic initiation, and the proportions of VLBW and ELBW infants with prolonged antibiotic duration. To assess changes in antibiotic use over the study period, we fit a logistic regression with a linear time variable (per quarter, defined as 3 calendar months) for each outcome (early antibiotic initiation and prolonged duration). From the models, we obtained expected risk (interpreted as proportion) for each quarter, and described the annual change of the expected risk (risk difference over 4 quarters). Confidence intervals for risk differences were calculated by the delta method. Robust standard errors were calculated to account for clustering at the hospital level. Analyses were performed with the full VLBW infant cohort as well as the subcohort of ELBW infants. Because not every center contributed patients throughout the study period, we also performed sensitivity analyses including (1) only those hospitals that contributed VLBW infants in all years and (2) only those hospitals that contributed ELBW infants in all years for respective analyses, to confirm that observed trends were not due to the effect of hospitals entering or leaving the cohort.

To describe variation of early antibiotic initiation and prolonged antibiotic duration by center, we used the most recent 12 months of the study period (October 2014 to September 2015). We chose this 12-month period to allow the most contemporary evaluation of variation, as well as to minimize the impact that changes in center-specific personnel and center-specific admission policies could have on antibiotic use. We excluded centers that admitted fewer than 10 VLBW or ELBW infants (for respective analyses) during the 12-month period because such centers are not likely to have consistent policies for the care of infants they care for rarely, and such centers are more likely to transfer VLBW and ELBW infants to higher-acuity centers in the 24 to 48 hours after birth.

A Wald test was used to determine the significance of the coefficient (*P* value) in the logistic regression models. *P* < .05 was considered statistically significant, and all reported *P* values are 2-sided. Statistical analyses were performed using Stata statistical software version 15.0 (StataCorp).

## Results

### Characteristics of the Study Participants and Centers

**Figure 1.**
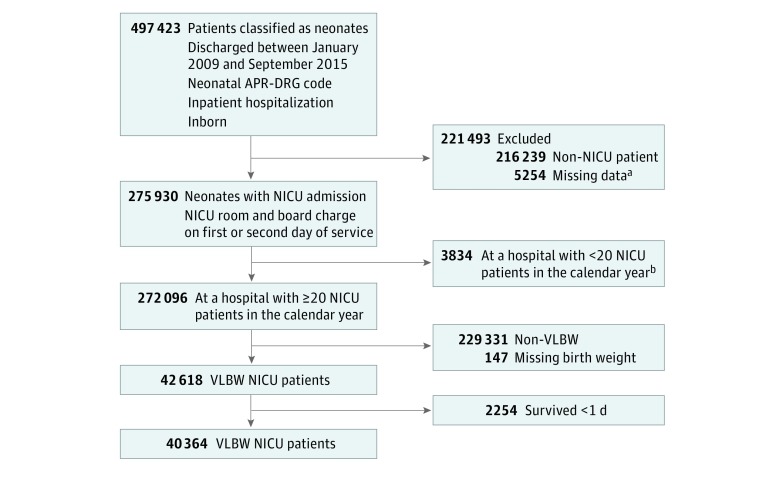
Patient Flow Diagram APR-DRG indicates All Patient Refined Diagnosis Related Group; NICU, neonatal intensive care unit; VLBW, very low-birth-weight. ^a^Patient did not have any services billed in the first 3 days of admission. ^b^For the year 2015, which only includes 3 quarters, patients at hospitals with fewer than 15 NICU patients were excluded.

We identified 42 618 VLBW infants (Figure 1), of whom 40 364 (94.7%) survived for at least 1 day, including 14 923 ELBW infants, of whom 12 947 (86.8%) survived for at least 1 day. Female infants accounted for 50.7% (20 447 of 40 364) of the study participants. The number of infants included per quarter ranged from 1257 to 1676 VLBW infants and 410 to 556 ELBW infants. Of the 297 centers contributing VLBW infants, most had more than 200 total inpatient beds (254 [85.5%]), were not teaching centers (183 [61.6%]), and were located in urban areas (265 [89.2%]). Geographically, all regions of the country were represented in the sample: 135 centers (45.5%) were located in the US South, 60 (20.2%) in the Midwest, 56 (18.8%) in the West, and 46 (15.5%) in the Northeast.

### Antibiotic Initiation and Prolonged Administration

**Figure 2.**
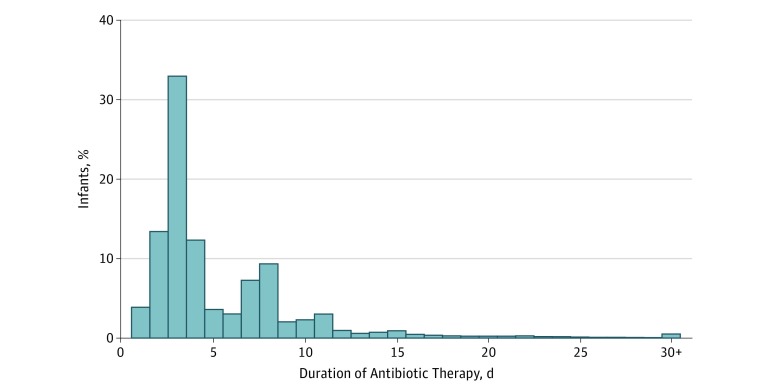
Duration of Early Antibiotic Therapy The graph includes very low-birth-weight infants who received antibiotic therapy starting in the first 3 days, were admitted to the hospital, and survived for at least 5 days.

Antibiotics were initiated within 3 days of age for 31 715 VLBW infants (78.6%) and 11 264 ELBW infants (87.0%). Infants administered early antibiotic therapy had lower birth weights, were less likely to be cared for in teaching centers, and had longer lengths of stay compared with those not administered early antibiotic therapy (Table). Ampicillin (74%) and gentamicin (68%) were the most commonly administered antibiotics, while broader-spectrum antibiotics (cefotaxime, 5%; cefepime, 2%; vancomycin, 1%) were less frequently initiated. Among infants who survived and remained in the NICU for more than 5 days, 10 200 of 38 542 VLBW infants (26.5%) and 4521 of 11 977 ELBW infants (37.8%) received prolonged antibiotic therapy; durations of therapy for the VLBW infants are shown in Figure 2.

**Table.** Unadjusted Comparison of Very Low-Birth-Weight Infant Demographic Characteristics

Characteristic	Infants (N = 40 364)	
	Early Antibiotic Initiation (n = 31 715)	No Early Antibiotic Initiation (n = 8649)
Female, No. (%)	15 632 (49.3)	4815 (55.7)
Birth weight <750 g, No. (%)	4897 (15.4)	549 (6.3)
Admitted to teaching hospital, No. (%)	19 465 (61.4)	5710 (66.0)
Admitted in urban setting, No. (%)	30 603 (96.5)	8432 (96.5)
Length of stay, median (IQR), d	52 (32-77)	35 (23-52)

Abbreviation: IQR, interquartile range.

**Figure 3.**
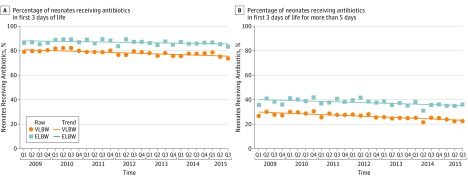
Temporal Trend Analyses of Antibiotic Initiation and Continuation From 2009 to 2015 A, There were no significant differences in early antibiotic initiation rates over time for very low-birth-weight (VLBW) and extremely low-birth-weight (ELBW) infants. B, There was a statistically significant decrease over time in the rate of prolonged antibiotic duration for VLBW infants but not for ELBW infants. A and B, The VLBW cohort includes ELBW infants. Q indicates quarter.

The raw proportion of infants administered early antibiotic therapy ranged from 73.9% to 82.5% per quarter for VLBW infants and from 83.6% to 89.6% per quarter for ELBW infants. Based on the fitted models, there were no significant differences in early antibiotic initiation rates over time for both populations (*P* = .12 for VLBW; *P* = .52 for ELBW) (Figure 3A). The annual risk difference in the proportion of VLBW infants administered early antibiotic therapy ranged from −0.75% (95% CI, −1.61% to 0.11%) to −0.87% (95% CI, −2.04% to 0.30%); in ELBW infants the annual risk difference ranged from −0.34% (95% CI, −1.28% to 0.61%) to −0.38% (95% CI, −1.61% to 0.85%).

The raw proportion of infants with prolonged antibiotic duration ranged from 21.5% to 30.5% per quarter for VLBW infants and 31.0% to 41.9% per quarter for ELBW infants. Based on the fitted models, there was a statistically significant decrease over time in the rate of prolonged antibiotic duration for VLBW infants (*P* = .02) but not for ELBW infants (*P* = .22) (Figure 3B). The annual risk difference in the proportion of VLBW infants with prolonged antibiotic duration ranged from −0.94% (95% CI, −1.65% to −0.23%) to −1.08% (95% CI, −2.00% to −0.16%); in ELBW infants the annual risk difference ranged from −0.72% (95% CI, −1.83% to 0.39%) to −0.75% (95% CI, −1.96% to 0.46%).

Sensitivity analyses including only those hospitals that contributed VLBW or ELBW infants in all years for respective analyses were consistent with the primary analyses. The annual risk difference in the proportion of VLBW infants administered early antibiotic therapy ranged from −1.00% (95% CI, −1.96% to −0.04%) to −1.26% (95% CI, −2.81% to 0.29%) (*P* = .08); in ELBW infants the annual risk difference ranged from −0.68% (95% CI, −1.83% to 0.46%) to −0.91% (95% CI, −3.00% to 1.17%) (*P* = .35). The annual risk difference in the proportion of VLBW infants with prolonged antibiotic duration ranged from −1.14% (95% CI, −2.11% to −0.17%) to −1.01% (95% CI, −1.77% to −0.24%) (*P* = .01); in ELBW infants the annual risk difference ranged from −1.00% (95% CI, −2.33% to 0.34%) to −0.95% (95% CI, −2.18% to 0.27%) (*P* = .14).

### Variation Analysis

**Figure 4.**
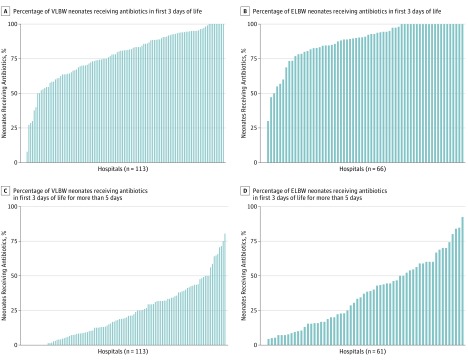
Variation Analysis of Early Antibiotic Use Across Centers Sixty-nine of 113 centers (61.1%) started antibiotic therapy for more than 75% of very low-birth-weight (VLBW) infants, and 56 of 66 centers (84.8%) started antibiotic therapy for more than 75% of extremely low-birth-weight (ELBW) infants. The graphs reflect data from a 12-month period comprising the fourth quarter of 2014 to the third quarter of 2015. A and B, Variation in antibiotic initiation was noted across centers for both VLBW and ELBW infants. C and D, Even greater variation was observed across centers in prolonged antibiotic administration, particularly among ELBW infants. A and C, Hospitals with fewer than 10 VLBW neonates were excluded. B and D, Hospitals with fewer than 10 ELBW neonates were excluded.

Sixty-nine of 113 centers (61.1%) started antibiotic therapy for more than 75% of VLBW infants, and 56 of 66 centers (84.8%) started antibiotic therapy for more than 75% of ELBW infants. Variation in antibiotic initiation was noted across centers (Figure 4A and B). Compared with antibiotic initiation, there was more substantial variation across centers in prolonged antibiotic administration, particularly among ELBW infants (Figure 4C and D). The proportion of VLBW and ELBW infants administered prolonged antibiotics ranged from 0% to 80.4% and 0% to 92.0% across centers, respectively.

## Discussion

We examined early antibiotic administration among VLBW infants admitted to nearly 300 NICUs across the United States and observed high rates of antibiotic exposure during the first days after birth. Although VLBW premature infants are at higher risk of early-onset infection compared with term-born infants, we found an overall rate of antibiotic initiation that was an order of magnitude higher than the actual incidence of infection^5,29,30^ and little change in antibiotic use over a contemporary 7-year period. Most notably, we observed wide variation among centers in use of antibiotics for both VLBW and ELBW infants. These observations identify several issues that should be addressed to optimize antibiotic stewardship among VLBW infants.

This analysis represents the largest study to our knowledge of early antibiotic use among VLBW infants across the United States and included both academic and community hospitals. Smaller studies have addressed early antibiotic exposures among VLBW and/or ELBW infants in the United States, namely within academic centers, over the past 20 years. A study conducted among 4039 ELBW infants born in 19 Neonatal Research Network centers from 1998 to 2001 found that 96% were administered empirical antibiotics within 72 hours of birth, and 53% continued to receive these antibiotics for 5 or more days in the absence of culture-confirmed infection.^8^ Another study included 790 ELBW infants born in 2000 at multiple centers within a university health care system in the United States; 94% were administered early empirical antibiotics, and 59% of those with negative blood cultures continued to receive antibiotics for 4 or more days.^7^ In a more recent analysis of antibiotic use rates among VLBW infants born from 2010 to 2014 in the Canadian Neonatal Network, 55% of 8414 VLBW infants without infection-related comorbidities received antibiotics for 4 or more days during their first 7 days after birth.^12^ The Centers for Disease Control and Prevention’s 12-Step Program to Prevent Antimicrobial Resistance in Health Care Settings was released in 2002.^31^ Patel and colleagues^32^ demonstrated that the antimicrobial stewardship elements of this campaign were relevant yet not frequently applied in their study of 4 tertiary care NICUs in 2005. A decade later, our study found overall lower rates of both antibiotic initiation and prolonged antibiotic administration among VLBW and ELBW infants compared with prior reports, potentially reflecting increased attention to indiscriminate antimicrobial use in the premature population. However, we found little change over the past 7 years, reflecting continued uncertainty among neonatal clinicians about the optimal application of antibiotic stewardship principles among very premature infants.

The substantial rates of antibiotic initiation observed in this study are not surprising given the frequency of clinical instability among VLBW and ELBW infants^33^ and national guidance that has advocated the use of antibiotics for critical illness.^34^ The wide site variation in administration of prolonged antibiotics likely reflects clinical uncertainty more than variation in illness severity between sites, particularly among ELBW infants, who almost universally manifest some degree of cardiorespiratory instability. Significant site variation in antibiotic use rates was also noted in a study of 127 NICUs in California.^19^ This study analyzed infants of all gestational ages throughout their hospitalization and observed 40-fold variation independent of confirmed infection and infection-related morbidities and mortality. Little attention has been given to the identification of premature newborns who might be spared early antibiotic exposure. Two recent studies used delivery characteristics to identify VLBW and ELBW infants at lower risk of EOS.^35,36^ One study that analyzed all cases of EOS occurring among 5313 VLBW infants over a 25-year period at a single high-risk neonatal center found that 97% of the 109 identified cases occurred in infants born with some combination of premature rupture of membranes, preterm labor, or concern for intrauterine infection.^35^ In another study of 15 318 infants born at 22 to 28 weeks’ gestation at multiple centers within the Neonatal Research Network, those born by cesarean delivery with membrane rupture at delivery and without clinical chorioamnionitis were significantly less likely to have confirmed EOS.^36^ When placental pathology results were available, the additional absence of histologic chorioamnionitis defined ELBW infants at even lower risk of EOS. It is therefore possible that clinical strategies based on such delivery characteristics and/or placental pathologic findings might provide a framework for developing approaches to decreasing empirical antibiotic use among VLBW and ELBW infants.

### Limitations

Our study has several limitations. The Premier database does not contain information that allows for the calculation and comparison of scores for early illness severity, so we cannot directly address the role that local variation in patient acuity contributed to antibiotic use variation. In addition, neither microbiologic information nor other indications for antibiotic therapy were available, so we could not identify infants with culture-confirmed infections. While all regions of the country were represented in the center sample, the overrepresentation of centers located in the southern United States should be taken into consideration when interpreting these results. A small number of centers in the variation analysis have very low antibiotic use, which may be due to true differences in practice, patient population, or center-specific incidence of culture-confirmed infection or to errors in billing charge entry.

## Conclusions

Most VLBW and ELBW infants receive empirical early antibiotic therapy, and this has remained stable over time. There is discordance between rates of early antibiotic exposure and incidence of EOS in premature infants, and variation in exposure rates exists across centers. These findings suggest a continued need for neonatal antibiotic stewardship efforts designed to help clinicians identify premature infants at lowest risk of EOS to avoid nonindicated, and perhaps harmful, antibiotic exposure.
